# Notes and News

**Published:** 1900-09-15

**Authors:** 


### Notes and News.

The Middlesex Hospital has received a donation of 1,000 guineas from Miss Wicksteed to endow a bed in perpetuity in the cancer ward for female patients.

Major Hamil Frith, F.R.C.S., R.A.M.C., has been appointed Professor of Military Hygiene at the Army Medical School, Netley, in place of Dr. Notter, resigned.

Two fresh cases of plague are reported at Glasgow, one coming from the east and the other from the south side of the city. A further number of persons have, in consequence, been placed under observation.

A lady has been appointed as medical officer at the Dundee East Poorhouse in the person of Dr. Laura Sandeman, who pursued her medical studies at Dundee, Edinburgh, and Dublin.

A new method for the treatment of hay fever is being practised in New York. The patient is placed in a cold storage for some hours, and relief is said to result. Already plans for establishments where this cold treatment can be carried out are being contemplated.

The annual report of the principal chemist of the Government Laboratory has just been issued as a Parliamentary paper. A large number of the samples of imported butter contained boric preservatives and were artificially coloured, and, as usual, it was found that the use of these preservatives was most prevalent in France, Belgium, and Australia, and was very common also in Holland. The most frequent colouring matter was annatto, but the use of coal tar yellows appeared to be on the increase. The bulk of the margarine imported came from Holland. It was usually made with cotton-seed oil, contained boric preservative, and was artificially coloured with a coal tar yellow. The fact is that without some such constant supervision as is exercised by the various authorities, adulteration would run unrestricted riot among food products. Even as it is, 13 per cent. of the beers analysed were found to have been diluted or otherwise adulterated, and nearly a quarter of the samples of tobacco taken from the manufacturers and dealers were found to be adulterated with liquorice or glycerine. Alas, for honesty! Where is it to be found?

The Sanitary Council in Constantinople has enjoined all persons to cover their feet, that they may not become infected by any abrasions of the feet, and thus be the means of spreading disease.

The Congress of German naturalists and medical men, which is holding its annual meeting this week at Aix-la-Chapelle, will be addressed by a number of leading scientific men speaking the German language. The discussions extend over a wide range of subjects, including physics, chemistry, biology, anatomy, physiology, pathology, medicine, hygiene, balmeology, and numerous allied topics.

The Local Government Board has given its approval to a scheme to provide a new workhouse infirmary for Stockport. They have refused, however, to ratify a proposal that the expenditure per bed for the building should be limited to a figure proposed by a committee of the Guardians. As the Board of Guardians is very much divided upon the subject of expenditure it appears likely that Stockport may have to wait some time for its infirmary.

The Manchester City Council has at last decided to accept the plan for the disposal of their sewage which has been framed to meet the requirements of the Local Government Board. Both this scheme and that proposed by the experts engaged by the Corporation were based upon bacterial treatment, the important point of difference largely hinging upon the fact that the Government scheme involved the use of a much larger acreage for the subsequent treatment of the effluent. On the Government scheme being adopted, a resolution was passed to apply for the sanction of the Local Government Board to the borrowing of the £487,000 required to carry it out.

Referring to the correspondence which has lately taken place regarding the prevalence of lung diseases in the navy, Dr. Mitchell Bruce states that the loss of useful life from tuberculosis is probably three times as great in training ships for boys in our navy as in the general population; and, as he justly says, this becomes all the more startling when we consider that the boys whose lives are thus wrecked by tuberculosis have been admitted to the navy after a strict medical test only a year or two previously. In seeking for the cause of this condition of affairs one's suspicions naturally fall upon the quarters in which the boys live. Dr. Mitchell Bruce, comparing the returns of the health of the boys in a wooden ship and in an iron ship respectively, finds that the invalidings and deaths in the "Black Prince" (iron) were 2·812 per cent., while in the "Impregnable" (wooden) they were 4·660 per cent. These figures do not relate specially to tuberculosis, but from private information he learns that the most common cause of invaliding and death in the training ships is diseases of the lungs and pleura. He has also been informed that the old wooden ships, such as the "Impregnable," are overcrowded, low-decked, wretchedly ventilated, and full of decay. Undoubtedly in the present state of our knowledge in regard to the causation of tuberculosis the conditions mentioned by Dr. Mitchell Bruce ought to be remedied without delay.

